# Artificial intelligence-enabled screening strategy for drug repurposing in monoclonal gammopathy of undetermined significance

**DOI:** 10.1038/s41408-023-00798-7

**Published:** 2023-02-17

**Authors:** Alexander J. Ryu, Shaji Kumar, Angela Dispenzieri, Robert A. Kyle, S. Vincent Rajkumar, Thomas C. Kingsley

**Affiliations:** 1 Division of Hospital Internal Medicine, Mayo Clinic, Rochester, MN USA; 2 Division of Hematology, Mayo Clinic, Rochester, MN USA; 3 Department of Quantitative Health Sciences, Rochester, MN USA

**Keywords:** Translational research, Drug development

## Abstract

Monoclonal gammopathy of undetermined significance (MGUS) is a benign hematological condition with the potential to progress to malignant conditions including multiple myeloma and Waldenstrom macroglobulinemia. Medications that modify progression risk have yet to be identified. To investigate, we leveraged machine-learning and electronic health record (EHR) data to screen for drug repurposing candidates. We extracted clinical and laboratory data from a manually curated MGUS database, containing 16,752 MGUS patients diagnosed from January 1, 2000 through December 31, 2021, prospectively maintained at Mayo Clinic. We merged this with comorbidity and medication data from the EHR. Medications were mapped to 21 drug classes of interest. The XGBoost module was then used to train a primary Cox survival model; sensitivity analyses were also performed limiting the study group to those with non-IgM MGUS and those with M-spikes >0.3 g/dl. The impact of explanatory features was quantified as hazard ratios after generating distributions using bootstrapping. Medication data were available for 12,253 patients; those without medications data were excluded. Our model achieved a good fit of the data with inverse probability of censoring weights concordance index of 0.883. The presence of multivitamins, immunosuppression, non-coronary NSAIDS, proton pump inhibitors, vitamin D supplementation, opioids, statins and beta-blockers were associated with significantly lower hazard ratio for MGUS progression in our primary model; multivitamins and non-coronary NSAIDs remained significant across both sensitivity analyses. This work could inform subsequent prospective studies, or similar studies in other disease states.

## Introduction

Monoclonal gammopathy of undetermined significance (MGUS) is currently understood as a benign hematological disease with the potential to progress to malignant conditions including multiple myeloma and light chain amyloidosis. While previous research has identified certain laboratory findings that are predictive of MGUS progression risk, there are currently no medications identified that can decrease progression risk [1]. While recent phase 3 trials have shown benefit in patients with smoldering multiple myeloma, there have not been any data supporting intervention in the MGUS phase given the low risk of progression and the toxicity associated with therapies used for multiple myeloma. More recently, clinical trials are underway studying daratumumab, cancer vaccines and rifaximin for early intervention in MGUS [2].

In addition to traditional clinical trials, drug repurposing may offer another strategy for discovering potential treatments for conditions where none exist, such as MGUS. Drug repurposing studies may be conducted prospectively, or using various forms of retrospective, real-world data such as claims or electronic health record (EHR) data. Drug repurposing studies performed on real-world data excel relative to clinical trials in terms of time to results and cost. Moreover, these studies allow us to identify drugs that have been used for common conditions with excellent safety record which can be leveraged for more benign conditions like MGUS. To date, there is limited published work on using real-world data to study drug repurposing for MGUS.

Here, we propose a method for using explainable machine learning on electronic medical records data to generate hypotheses about possible drug repurposing candidates in MGUS. We propose that the results of a screening analysis such as this could then be used to inform more in-depth studies such as synthetic clinical trials or traditional clinical trials. In particular, we leverage clinical data on a large cohort of MGUS patients, pair this with medications data, also obtained from the EHR, and use explainable machine-learning to search for associations between the medications patients were taking for other indications, and MGUS progression.

## Patients and methods

### Data

This study was approved by the Mayo Clinic Institutional Review Board (# 14-004382). Patient data were used under a waiver of consent. We extracted MGUS clinical and laboratory data from a manually curated MGUS database, prospectively maintained at Mayo Clinic. This database contained 16 752 MGUS patients, diagnosed from January 1, 2000, through December 31, 2021, who were seen at Mayo Clinic. Additionally, we extracted medication data from our EHR, which we linked to patients’ clinical and laboratory data. Medications were then coded by drug class; we were not able to reliably ascertain patients’ medication quantities from our EHR, so only the presence or absence of a medication from a given class was recorded. Drug class mappings are described in Supplementary Table 1. Only medication exposure prior to progression from MGUS to myeloma or a related condition was included for those patients with a documented progression. In cases where treatment with a given medication class could be used as treatment for an MGUS progression disease state, such as rituximab for Waldenstrom Macroglobulinemia, medical records were manually reviewed to ensure this was not a mislabeled progression event. Finally, we also extracted patients’ major comorbidities from our EHR and linked these data with the MGUS database. Comorbidities were defined as the presence of a diagnosis code within a patient’s record, between the time of diagnosis and any survival endpoint, corresponding to any of chronic heart disease (heart failure of any type or coronary artery disease), chronic kidney disease, chronic liver disease (cirrhosis or fatty liver disease) or diabetes mellitus (any subtype). Data were then split randomly into training/validation/test sets using a 70/15/15 percentage split.

### Primary analysis

We then fit a gradient-boosted machines Cox survival model to the data, where survival was defined as the time from MGUS diagnosis to progression to multiple myeloma, Waldenström macroglobulinemia or systemic amyloidosis. In particular, we used the XGBoost package with “Cox” training objective [3]. After fitting the model, we computed the concordance index of test set predictions using the inverse probability of censoring weights function from scikit-survival [4]. Finally, we used bootstrapping and Shapley Additive Explanation values to estimate hazard ratios and their distributions for each of the model features, as described in a recent manuscript [5, 6].

### Sensitivity analyses

We additionally performed sensitivity analyses in which we limited the study group to those with an M spike >0.3 g/dl and those with non-IgM MGUS. We have reported the results of these sensitivity analyses separately.

## Results

Our MGUS database contained 16,752 patients; medication data were only available for 12,253 of these patients. Demographics, clinical and laboratory values for both the study group and the group excluded due to a lack of medication data are displayed in Table 1. We did not observe any significant differences between the cohort included for the analysis and the patients excluded from the study.

**Table 1:** Study cohort and no-medication cohort characteristics.

	Study cohort	No-medication cohort	*P*-value
Demographics			
* N*	12 253	4 471	
% female	41.2	41.6	*P* = 0.6
Age (mean ± SD)	67.6 ± 12.3 years	68.1 ± 12.7 years	*P* = 0.97
Follow-up (median, 95% CI)	2.07 years (0.09–13.1 years)	0.94 years (0.006–9.0 years)	Log-rank *P* < 0.005
Caucasian	92.40%	91.80%	*P* = 0.2
Black	2.00%	1.80%	*P* = 0.4
Asian	0.90%	0.70%	*P* = 0.2
American Indian	0.40%	0.40%	*P* = 1
Pacific Islander	0.05%	0.07%	*P* = 0.6
Other	1.30%	1.20%	*P* = 0.6
Unknown	2.60%	3.20%	*P* < 0.05
Clinical data			
Chronic heart disease	16.90%	4.60%	*P* < 0.00001
Chronic kidney disease	23.30%	12.10%	*P* < 0.00001
Chronic liver disease	6.40%	3.80%	*P* = 1
Diabetes	23.10%	19.10%	*P* < 0.00001
BMI	29.0 ± 12.2	28.5 ± 10.8	*P* < 0.05
Died %	22.60%	38.90%	*P* < 0.00001
Progression outcome %	3.30%	2.40%	*P* < 0.05
Laboratory data			
Serum M-protein (mean ± SD, *n*)	0.33 ± 0.56 g/dL, 10,254	0.34 ± 0.52 g/dL, 3,384	*P* = 0.9
Free light chain ratio (mean ± SD, *n*)	4.92 ± 62.3, 5,382	3.63 ± 19.6, 361	*P* = 0.9
Serum IgA subtype	12.20%	12.30%	*P* = 0.9
Serum IgM subtype	23.00%	24.50%	*P* < 0.05
Serum IgG subtype	63.60%	61.10%	*P* < 0.01
Other Ig subtype	1.20%	2.10%	*P* < 0.01

We then examined the relationship between different drug exposure and the risk of progression from MGUS. Within the study cohort, the frequency of medication usage by progression outcome is shown in Table 2.

**Table 2:** Frequency of medication usage by progression outcome.

	Progression (*n* = 404)	No progression (*n* = 11849)	*P*-value
Medication class			
Anticoagulant	16.20%	22.70%	*P* < 0.00001
ACE inhibitor	31.30%	36.60%	*P* < 0.00001
Beta blocker	31.60%	45.10%	*P* < 0.00001
Myelodysplastic syndrome drug	5.80%	0.90%	*P* < 0.00001
NSAID	18.00%	22.10%	*P* < 0.00001
Proton-pump inhibitor	27.60%	43.10%	*P* < 0.00001
Antiplatelet drug	48.40%	43.80%	*P* < 0.00001
Estrogen supplement	1.80%	1.70%	*P* = 0.7
Folic acid	15.50%	20.50%	*P* < 0.00001
Aldosterone receptor antagonist	7.80%	10.90%	*P* < 0.00001
Immunomodulator	4.30%	9.80%	*P* < 0.00001
Loop diuretic	20.60%	28.80%	*P* < 0.00001
Metformin	7.80%	11.30%	*P* < 0.00001
Multivitamin	30.80%	42.10%	*P* < 0.00001
Opioid	22.10%	34.30%	*P* < 0.00001
Rituximab	1.50%	1.60%	P = 0.6
Statin	35.30%	49.70%	*P* < 0.00001
Corticosteroid	20.80%	30.20%	*P* < 0.00001
Testosterone supplement	1.80%	2.10%	*P* = 0.2
Thyroid replacement	15.00%	19.70%	*P* < 0.00001
Vitamin D supplement	46.40%	49.60%	*P* < 0.001

The study cohort was then randomly split into training, validation, and test sets with 8,577 patients in the training set, and 1 838 patients in each of the validation and test sets. The inverse probability of censoring weights (IPCW) concordance index was calculated for the test set at 0.883, indicating good model fit.

Finally, the associated hazard ratios for MGUS progression of each model feature are shown in Table 3. Both clinical and medication features are shown. As expected, higher baseline serum M-spike and body mass index were associated with significantly higher odds of MGUS progression. Within the medication groups examined, the presence of multivitamins, immunosuppression, non-coronary NSAIDS, proton pump inhibitors, vitamin D supplementation, opioids, statins, and beta-blockers were associated with significantly lower odds of MGUS progression.

**Table 3:** Model features and associated hazard ratios with confidence intervals.

Model feature	Median HR	5th percentile HR	95th percentile HR
Patient characteristics (statistically significant)			
Death from other cause	0.0541	0.0286	0.0788
Chronic liver disease	0.472	0.214	0.821
Chronic heart disease	0.484	0.323	0.75
Solid organ transplant	0.577	0.217	0.983
Body mass index	1.808	1.169	2.821
Disease characteristics (statistically significant)			
Serum IgM level	0.519	0.303	0.834
Hemoglobin	0.554	0.35	0.931
IgG subtype	0.621	0.415	0.856
Calcium	0.633	0.406	0.946
IgM subtype	1.618	1.106	2.561
IgA subtype	1.814	1.24	2.7
Serum M-spike	4.931	2.908	10.245
Medications (statistically significant)			
Multivitamin	0.495	0.345	0.706
Immunosuppression	0.589	0.289	0.956
Non-coronary NSAID	0.614	0.412	0.856
Proton pump inhibitor	0.626	0.429	0.889
Vitamin D supplementation	0.675	0.47	0.928
Opioid	0.676	0.471	0.935
Statin	0.682	0.48	0.973
Beta blocker	0.742	0.521	0.972
Patient characteristics (non-significant)			
Black race	0.497	0.194	1
Diabetes	0.743	0.497	1.064
Autoimmune disease	0.789	0.386	1.3
White race	0.88	0.535	1.642
History of bacteremia	1	0.802	1.345
American Indian race	1	1	1
Pacific Islander race	1	1	1
Other race	1	1	1
Asian race	1	0.965	9.281
Race Unknown	1	1	1
Chronic kidney disease	1.067	0.674	1.639
Male	1.254	0.921	1.754
Age at diagnosis	1.442	0.802	2.285
Disease characteristics (non-significant)			
Serum IgA level	0.713	0.441	1.106
Serum IgG level	0.768	0.463	1.384
Serum albumin level	0.889	0.584	1.365
Serum creatinine	0.972	0.587	1.622
Beta 2 glycoprotein	1.035	0.612	1.986
Serum kappa level	1.12	0.699	2.063
Serum kappa/lambda ratio	1.4227	0.88	2.611
Serum lambda level	1.504	0.88	2.611
Medications (non-significant)			
Testosterone supplementation	0.673	0.223	1.295
Thyroid supplementation	0.715	0.471	1.03
Folic acid	0.747	0.502	1.048
ACE inhibitor	0.782	0.54	1.105
Metformin	0.848	0.488	1.296
Rituximab	0.85	0.369	1
Aldosterone antagnoist	0.915	0.554	1.303
Estrogen supplementation	1	0.663	1.668
Corticosteroid	1.014	0.678	1.412
Therapeutic dose anticoagulant	1.073	0.759	1.429
Antiplatelet medication	1.119	0.799	1.522
Loop diuretic	1.299	0.926	1.801
Myelodysplastic syndrome drug	1.858	0.722	5.174

With respect to sensitivity analyses, we repeated the above methodology for study groups limited to those with only an M spike >0.3 g/dl and those with IgM MGUS excluded. 3 507 patients had an M spike >0.3 g/dl, 10 333 patients had non-IgM MGUS subtypes. An analogous model training process fit models to each of these four data sets. The IPCW concordance index for each model was: 0.817 for the high M spike model, 0.889 for the non-IgM model. In the high-M spike model, the presence of multivitamins, non-coronary NSAIDs, and metformin were associated with significantly lower odds of MGUS progression. In the non-IgM model, multivitamins, immunosuppression, non-coronary NSAIDs, statins, proton pump inhibitors, and opioids were associated with significantly lower odds of progression, while loop diuretics were associated with an increased risk of progression. The medications with statistically significant hazard ratios in each model are shown in Supplementary Fig. 3.

## Discussion

In this manuscript, we describe a method for generating drug repurposing hypotheses in MGUS using EHR data and explainable machine-learning. We accomplished this using an XGBoost Cox survival model and Shapley feature explanations. Our model achieved an adequate fit of the survival data.

This study represents the first application of machine-learning for screening drug repurposing candidates in MGUS. We propose this methodology as a low-cost precursor prior to examining drug candidates of interest in synthetic clinical trials or prospective trials. Completing this type of study from EHR data of course requires access to a relatively large cohort of patients with a given disease and fairly complete follow-up data, both of which were available due to our status as an academic tertiary referral center.

When examining feature hazard ratios shown in Table 3, several medication classes appeared to be associated with reduced odds of MGUS progression, specifically, multivitamins, immunosuppression, non-coronary NSAIDS, proton pump inhibitors, vitamin D supplementation, opioids, statins, and beta-blockers. The literature review did not reveal any known associations between multivitamin, NSAID, or opioid use and MGUS progression. Regarding immunosuppression, tacrolimus, cyclosporine, and methotrexate were included. The literature review suggested that in both renal transplant and liver transplant populations, there was no association between the tacrolimus versus cyclosporine-based immunosuppression regimens and the development of MGUS or MGUS progression outcomes, though the number of such outcomes was small [7–9]. No studies reported an association between methotrexate use and MGUS progression risk. Overall, it is possible that this protective association with immunosuppression may reflect the fact that patients on these drugs typically undergo extensive laboratory evaluations for their comorbidities, and thus a greater number of benign MGUS cases are detected relative to the broader population.

With respect to proton pump inhibitors, our analysis also suggested a significant protective association with proton pump inhibitors as well, a finding that has not previously been reported. There have, however, been reports of progressive intestinal microbiome disturbances in patients with MGUS and multiple myeloma, compared to normal patients [10]. Proton pump inhibitors are known to modify the intestinal microbiome, in ways generally thought to be deleterious; however, our finding of a positive association with MGUS outcomes may warrant further investigation [11]. With respect to Vitamin D supplementation use, prior research has demonstrated significantly lower levels of vitamin D2 and provitamin D3 in Waldenström Macroglobulinemia patients relative to IgM MGUS patients [12]. A causative role has not yet been established, however.

Limited research has been devoted to any association between statins and MGUS progression, but one letter to the editor reported no relationship between statins and MGUS progression in a 200-patient case-control study, while another study reported an association between statin use and improved multiple myeloma survival in a cohort study [13, 14]. Additionally, a network meta-analysis has demonstrated an all-cause mortality benefit with statin use among patients with or at risk for cardiovascular disease [15]. With respect to beta-blockers, a prior retrospective cohort study noted better outcomes among multiple myeloma patients on beta-blockers; similar results in MGUS patients have not previously been reported. Regarding thyroid supplementation, at least one population-based study, which included 19,303 patients with MGUS, noted a lower risk of disease progression in patients with autoimmune disease; the association between lower progression risk and the presence of thyroid supplementation may reflect this [16]. Overall, it is notable that, while associations between MGUS outcomes and metformin use appear to have been the most deeply explored in the literature to date [17–19], our primary analysis notes a stronger association with multivitamins immunosuppression, proton pump inhibitors, NSAIDS, Vitamin D supplementation, opioids, statins, and beta-blockers. Additionally, our sensitivity analysis limited to those with high M spike suggested that multivitamins, non-coronary NSAIDs, and metformin were associated with lower odds of MGUS progression. Analogously, our sensitivity analysis excluding those with IgM MGUS suggested that multivitamins, immunosuppression, non-coronary NSAIDs, statins, PPIs, and opioids were associated with lower odds of MGUS progression, while loop diuretics were associated with higher odds of progression.

Regarding non-medication feature hazard ratios, we note that body mass index and serum M-spike levels were associated with significantly higher odds of MGUS progression, consistent with existing literature [20].

### Limitations

Our study was limited by the inherent shortcomings of using her data to ascertain outcomes and medications. Detection of disease progression outcomes may be incomplete. We also note that our MGUS database may contain patients with small M spikes associated with autoimmune diseases, even though these may not represent clones with the potential to progress to malignant disease. We attempted to mitigate this through sensitivity, analysis, however. Medication data also may be incomplete, and we were unable to ascertain medication adherence or prescription durations. This may have contributed to the large proportion of patients taking corticosteroids, which may have been for short durations in some cases. In future studies, medication data could be enriched by linking to claims data. In our study, we were unable to obtain medication data for 27% of patients either because the medication history was not entered into the EHR or because the patients were not taking any medications. Demographic and laboratory characteristics between the two groups, nonetheless, were similar. As expected, the no-medication group had significantly fewer medical comorbidities. Finally, our study is also limited by its retrospective nature, limiting any inferences about causality.

In conclusion, we analyzed EHR data on a large cohort of MGUS patients using explainable machine-learning to examine associations between patients’ medications and their MGUS outcomes. We uncovered associations that have been previously suggested (better hematologic malignancy outcomes with statin and beta-blocker use) and others that have not (decreased risk of progression with proton pump inhibitor use). Of note, we detected the strongest associations among drugs that have received relatively less attention in related literature to date, namely, multivitamins, immunosuppression, proton pump inhibitors, and vitamin D supplementation. Future research should focus on prospectively investigating these associations and applying similar methodology to other disease states.

## Supplementary information

Reproducibility checklist (updated)

Supplement

## Data Availability

The datasets generated during and/or analyzed during the current study are available from the corresponding author upon reasonable request.

## Code availability

Our code is shared in Supplementary Appendix 2.
