# Supplementary material for: Artificial intelligence-enabled screening strategy for drug repurposing in monoclonal gammopathy of undetermined significance
Source: Blood Cancer J. 2023 Feb 17;13(1):28. doi: 10.1038/s41408-023-00798-7 (PMC9935510; doi:10.1038/s41408-023-00798-7)
Supplement: Supplementary file 2 — Supplement [file 41408_2023_798_MOESM2_ESM.docx]

| **Supplement 1: Drug class mapping** | |
| --- | --- |
| **Medication class** | **Drugs Included** |
| Anticoagulant | Any direct oral anticoagulant or low molecular weight heparin |
| ACE inhibitor | Any ACE inhibitor |
| Beta blocker | Any oral beta blocker |
| Myelodysplastic syndrome drug | Lenalidomide, Azacitadine, Decitabine |
| NSAID | Any oral formulation of ibuprofen, meloxicam, naproxen, diclofenac, celecoxib |
| Proton-pump inhibitor | Any oral proton pump inhibitor |
| Antiplatelet drug | Any oral formulation of aspirin, clopidogrel, prasugrel or ticagrelor |
| Estrogen supplement | Any oral estrogen supplement |
| Folic acid | Any oral folic acid formulation supplement |
| Aldosterone receptor antagonist | Any oral formulation of spironolactone or triamterene |
| Immunomodulator | Any oral formulation of mycophenolate, tacrolimus, cyclosporine or methotrexate |
| Loop diuretic | Any oral loop diuretic |
| Metformin | Oral metformin |
| Multivitamin | Oral multivitamin |
| Opioid | Oral opioids |
| Rituximab | Rituximab |
| Statin | Any oral statin formulation |
| Corticosteroid | Any oral corticosteroid |
| Testosterone supplement | Any oral testosterone formulation |
| Thyroid replacement | Oral levothyroxine |
| Vitamin D supplement | Any oral vitamin D formulation |

**Supplement 2: Python Code**

**# Label oral meds**

oralroutes = ['PO', 'ORAL', 'TUBE']

meds['oral'] = meds['route'].str.contains('|'.join(oralroutes))

**# Label meds by class**

BBs = ['olol', 'carvedilol', 'toprol', 'Toprol', 'coreg', 'Coreg', 'Tenormin', 'carvediloL', 'labetalol', 'atenoloL']

meds['BB'] = (meds['name'].str.contains('|'.join(BBs), case=False)==True) & (meds['oral']==1)

statins = ['statin', 'lipitor', 'Lipitor', 'Crestor', 'Zocor']

meds['statin'] = (meds['name'].str.contains('|'.join(statins), case=False)==True) & (meds['oral']==1)

steroids = ['olone', 'isone', 'iSONE', 'asone', 'isolone', 'budesonide']

meds['steroid'] = (meds['name'].str.contains('|'.join(steroids), case=False)==True) & (meds['oral']==1)

antiplt = ['aspirin', 'Aspirin', 'Ecotrin', 'ecotrin', 'Plavix', 'clopidogrel', 'Brilinta', 'ticagrelor', 'prasugrel', 'Effient']

meds['antiplt'] = (meds['name'].str.contains('|'.join(antiplt), case=False)==True) & (meds['oral']==1)

immod = ['mycophenolate', 'CellCept', 'Prograf', 'Myfortic', 'tacrolimus', 'cyclosporine', 'methotrexate']

meds['immod'] = (meds['name'].str.contains('|'.join(immod), case=False)==True) & (meds['oral']==1)

vitD = ['cholecalcif', 'vitamin D', 'Vitamin D', 'ergocal']

meds['vitD'] = (meds['name'].str.contains('|'.join(vitD), case=False)==True) & (meds['oral']==1)

meds['multivit'] = meds['name'].str.contains('multivit', case=False, regex=False) & (meds['oral']==1)

PPI = ['esomeprazole', 'pantoprazole', 'omeprazole', 'Prilosec', 'Protonix', 'Nexium']

meds['PPI'] = (meds['name'].str.contains('|'.join(PPI), case=False)==True) & (meds['oral']==1)

MDSdrug = ['Revlimid', 'lenalidomide', 'azacitadine', 'Vidaza', 'decitabine', 'Dacogen']

meds['MDSdrug'] = (meds['name'].str.contains('|'.join(MDSdrug), case=False)==True) & (meds['oral']==1)

NSAID = ['ibuprofen', 'Advil', 'Motrin', 'naproxen', 'Naprosyn', 'diclofenac', 'celecoxib', 'Celebrex', 'meloxicam']

meds['NSAID'] = (meds['name'].str.contains('|'.join(NSAID), case=False)==True) & (meds['oral']==1)

AC = ['warfarin', 'Coumadin', 'Jantoven', 'Xarelto', 'rivaroxaban', 'apixaban', 'Eliquis', 'enoxaparin', 'Lovenox', 'Pradaxa', 'dabigatran', 'fondaparinux', 'Arixtra']

meds['AC'] = (meds['name'].str.contains('|'.join(AC), case=False)==True) & (meds['oral']==1)

ACE = ['losartan', 'Losartan', 'lisinopril', 'Lisinopril', 'Cozaar', 'valsartan']

meds['ACE'] = (meds['name'].str.contains('|'.join(ACE), case=False)==True) & (meds['oral']==1)

thyroid = ['levothyroxine', 'Levothyroxine', 'synthroid', 'Synthroid']

meds['thyroid'] = (meds['name'].str.contains('|'.join(thyroid), case=False)==True) & (meds['oral']==1)

aldo = ['spironolactone', 'Spironolactone', 'Aldactone', 'aldactone', 'triamterene', 'Triamterene']

meds[‘aldo’] = (meds['name'].str.contains('|'.join(aldo), case=False)==True) & (meds['oral']==1)

meds['estrogen'] = meds['name'].str.contains('estrogen', case=False, regex=False)

meds['testosterone'] = meds['name'].str.contains('testosterone', case=False, regex=False)

meds['metformin'] = meds['name'].str.contains('metformin', case=False, regex=False)

folic = ['folic', 'folate', 'synthroid', 'Synthroid']

meds['folic'] = (meds['name'].str.contains('|'.join(folic), case=False)==True) & (meds['oral']==1)

opioid = ['oxycodone', 'oxycontin', 'hydromorphone', 'dilaudid', 'morphine', 'hydrocodone', 'norco', 'lortab', 'oxymorphone']

meds['opioid'] = (meds['name'].str.contains('|'.join(opioid), case=False)==True) & (meds['oral']==1)

loop = ['furosemide', 'lasix', 'torsemide', 'demadex', 'bumetanide', 'bumex']

meds['loop'] = (meds['name'].str.contains('|'.join(loop), case=False)==True) & (meds['oral']==1)

meds['ritux'] = meds['name'].str.contains('ritux', case=False, regex=False)

**# Label each medication as a drug class**

meds['drugclass'] = meds.iloc[:, 5:26].idxmax(axis=1)

**# Collapse to one row per MRN showing all drug classes the person took**

collapse_meds = meds.groupby('MRN', as_index=False).agg({'BB':'max', 'statin':'max', 'steroid':'max', 'antiplt':'max', 'immod':'max', 'vitD':'max', 'multivit':'max', 'PPI':'max', 'MDSdrug':'max', 'NSAID':'max', 'AC':'max', 'ACE':'max', 'thyroid':'max', 'aldo':'max', 'estrogen':'max', 'testosterone':'max', 'metformin':'max', 'folic':'max', 'opioid':'max', 'loop':'max', 'ritux':'max'})

**# Merge MGUS data and meds data**

merge1 = pd.merge(mgus, collapse_meds, how="left", on=['MRN'], validate='one_to_one', indicator=True)

**# Merge comorbidities data to MGUS+meds**

merge2 = pd.merge(merge1, comorbs, how="inner", on=['MRN'], validate='one_to_one')

**# Format survival and progression labels for XGBoost Cox package**

merge2['upper'] = merge2['Progression'].astype(int)*(np.inf)

merge2['lower'] = merge2['survival']

merge2.upper.fillna(merge2.lower, inplace=True)

X = merge2.drop(columns=['upper', 'lower', 'Progression', 'survival'])

y = merge2[['upper', 'lower']]

y_cox = np.where(merge2['Progression'] == True, merge2['lower'], -merge2['lower'])

**# Model cross-validation**

params_cox = {

# Parameters that we are going to tune.

'max_depth':7,

'min_child_weight': 8,

'eta':.1,

'subsample': 1,

'verbosity':1,

'colsample_bytree': 1,

#'num_class':3,

# Other parameters

'objective':'survival:cox',

'eval_metric':'cox-nloglik'

}

num_boost_round = 999

gridsearch_params = [

(max_depth, min_child_weight)

for max_depth in [3,5,8,12,15]

for min_child_weight in [2,5,10,12,15]

]

# Define initial best params and cox-nloglik

min_error = float("Inf")

best_params = None

for max_depth, min_child_weight in gridsearch_params:

print("CV with max_depth={}, min_child_weight={}".format(

max_depth,

min_child_weight))

# Update our parameters

params_cox['max_depth'] = max_depth

params_cox['min_child_weight'] = min_child_weight

# Run CV

cv_results = xgb.cv(

params_cox,

dtrain_cox,

num_boost_round=num_boost_round,

seed=42,

nfold=5,

metrics=params_cox['eval_metric'],

early_stopping_rounds=10

)

# Update best cox-nloglik

mean_error = cv_results['test-cox-nloglik-mean'].min()

boost_rounds = cv_results['test-cox-nloglik-mean'].argmin()

print("\tcox-nloglik {} for {} rounds".format(mean_error, boost_rounds))

if mean_error < min_error:

min_error = mean_error

best_params_dw = (max_depth,min_child_weight)

print("Best params_dw: {}, {}, error: {}".format(best_params_dw[0], best_params_dw[1], min_error))

params_cox.update({'max_depth':best_params_dw[0], 'min_child_weight': best_params_dw[1]})

print(params_cox)

num_boost_round = 999

min_error = float("Inf")

best_params = None

for eta in [.15, .1, .05]:

print("CV with eta={}".format(eta))

# We update our parameters

params_cox['eta'] = eta

# Run and time CV

cv_results = xgb.cv(

params_cox,

dtrain_cox,

num_boost_round=num_boost_round,

seed=42,

nfold=5,

metrics=params_cox['eval_metric'],

early_stopping_rounds=10

)

# Update best score

mean_error = cv_results['test-cox-nloglik-mean'].min()

boost_rounds = cv_results['test-cox-nloglik-mean'].argmin()

print("\terror {} for {} rounds\n".format(mean_error, boost_rounds))

if mean_error < min_error:

min_error = mean_error

best_params_eta = eta

print("Best params_eta: {}, error: {}".format(best_params_eta, min_error))

params_cox.update({'eta': best_params_eta})

print(params_cox)

num_boost_round = 999

gridsearch_params = [

(subsample, colsample)

for subsample in [i/10. for i in range(7,11)]

for colsample in [i/10. for i in range(7,11)]

]

min_error = float("Inf")

best_params = None

# We start by the largest values and go down to the smallest

for subsample, colsample in reversed(gridsearch_params):

print("CV with subsample={}, colsample={}".format(

subsample,

colsample))

# We update our parameters

params_cox['subsample'] = subsample

params_cox['colsample_bytree'] = colsample

# Run CV

cv_results = xgb.cv(

params_cox,

dtrain_cox,

num_boost_round=num_boost_round,

seed=42,

nfold=5,

metrics={'cox-nloglik'},

early_stopping_rounds=10

)

# Update best score

mean_error = cv_results['test-cox-nloglik-mean'].min()

boost_rounds = cv_results['test-cox-nloglik-mean'].argmin()

print("\terror {} for {} rounds".format(mean_error, boost_rounds))

if mean_error < min_error:

min_error = mean_error

best_params_sc = (subsample,colsample)

print("Best params_sc: {}, {}, error: {}".format(best_params_sc[0], best_params_sc[1], min_error))

params_cox.update({'subsample': best_params_sc[0], 'colsample_bytree': best_params_sc[1]})

print(params_cox)

num_boost_round = 999

**# Model training**

cox = xgb.train(

params_cox,

dtrain_cox,

num_boost_round=num_boost_round,

evals=[(dvalid_cox, "Valid")],

early_stopping_rounds=10

)

**# Calculate concordance index**

from sksurv.metrics import concordance_index_ipcw

concordance_index_ipcw(train_reformat, test_reformat, test['Preds'], tau=None, tied_tol=1e-08)

**# Bootstrapping**

X_features = X.columns

myHazRatios = [[] for i in range(len(X.columns))]

myIndexes = [i for i in range(len(X.columns))]

# print(myHazRatios_all, myIndexes_all)

myBinCols = [col for col in X.columns if all(X[col].value_counts().index.isin([0, 1]))]

myContCols = [col for col in X.columns if not all(X[col].value_counts().index.isin([0, 1]))]

print("My list of binary columns are :\n", myBinCols)

print("\nMy list of cont. columns are :\n", myContCols)

#bootstrap!

for i in range(1, 1001):

# sample with replacement:

X_train = Xy.sample(n = Xy.shape[0], random_state = i, replace = True) #sample w/ replacement num rows

y_train = X_train['y'] #y train are X_trains y column

X_train = X_train.drop(columns = ['y']) #X_train then needs to drop the y column

X_test = Xy.drop(X_train.index) #test features are the full - X_train

y_test = X_test['y'] #y test are X_tests y column

X_test = X_test.drop(columns = ['y']) #X_test then needs to drop they column

xgb_train = xgb.DMatrix(X_train, label=y_train)

xgb_test = xgb.DMatrix(X_test, label=y_test)

model_train = xgb.train(params_cox, xgb_train, 1000, evals = [(xgb_test, "test")], verbose_eval=False)

# let's get the SHAP vals and a HR from these based on our model

shap_values_full = shap.TreeExplainer(model_train).shap_values(X)

# let's build a list of HazRatios indexing by j columns

for j in range(len(myIndexes)):

curSHAP = shap_values_full[:, j]

curCol = X.columns[j]

# if continuous, split by median

if (curCol in myContCols):

myMed = X[curCol].median()

myHazRatios[j].append(np.mean(np.exp(curSHAP[X[curCol] >= myMed])) #changed to mean

/np.mean(np.exp(curSHAP[X[curCol] < myMed]))) #changed to mean

# else, split by 1 or 0

else:

myHazRatios[j].append(np.mean(np.exp(curSHAP[X[curCol] == 1])) #changed to mean

/np.mean(np.exp(curSHAP[X[curCol] == 0]))) #changed to mean

# Done!

# myHazRatios

# indexed by column index

X_features = X.columns

# create a list of lists of length 4: [[Median, Mean, LB, UB], [...]]

mySummaries = []

for i in range(len(myHazRatios)):

myHazRatios[i].sort()

mySummaries.append([np.median(myHazRatios[i]), np.mean(myHazRatios[i]),

myHazRatios[i][24], myHazRatios[i][974]])

# print([i for i in range(len(myHazRatios_all)) if X_features[i] in myContinuousCols])

print(mySummaries)

**Supplement 3: Statistically significant medication hazard ratios in sensitivity analyses**

High M spike model

| **Median HR** | **5^th^ percentile HR** | **95^th^ percentile HR** |  |
| --- | --- | --- | --- |
| 0.469 | 0.195 | 0.850 | **Multivitamin** |
| 0.528 | 0.115 | 0.993 | **Non-coronary NSAID** |
| 0.609 | 0.136 | 0.934 | **Metformin** |

No IgM MGUS model

| **Median HR** | **5^th^ percentile HR** | **95^th^ percentile HR** |  |
| --- | --- | --- | --- |
| 0.574 | 0.418 | 0.762 | **Multivitamin** |
| 0.589 | 0.278 | 0.965 | **Immunosuppression** |
| 0.632 | 0.449 | 0.865 | **Non-coronary NSAID** |
| 0.653 | 0.482 | 0.883 | **Statin** |
| 0.671 | 0.492 | 0.916 | **Proton pump inhibitor** |
| 0.706 | 0.499 | 0.963 | **Opioid** |
| 1.401 | 1.020 | 1.989 | **Loop diuretic** |
